# Behavioral Neuroadaptation to Alcohol: From Glucocorticoids to Histone Acetylation

**DOI:** 10.3389/fpsyt.2016.00165

**Published:** 2016-10-06

**Authors:** Nicole Mons, Daniel Beracochea

**Affiliations:** ^1^CNRS UMR 5287, Institut des Neurosciences cognitives et intégratives d’Aquitaine, Nouvelle Université de Bordeaux, Pessac, France

**Keywords:** alcoholism, epigenetic, learning and memory, glucocorticoid, anxiety, signaling, CREB, brain

## Abstract

A prime mechanism that contributes to the development and maintenance of alcoholism is the dysregulation of the hypothalamic–pituitary–adrenal axis activity and the release of glucocorticoids (cortisol in humans and primates, corticosterone in rodents) from the adrenal glands. In the brain, sustained, local elevation of glucocorticoid concentration even long after cessation of chronic alcohol consumption compromises functional integrity of a circuit, including the prefrontal cortex (PFC), the hippocampus (HPC), and the amygdala (AMG). These structures are implicated in learning and memory processes as well as in orchestrating neuroadaptive responses to stress and anxiety responses. Thus, potentiation of anxiety-related neuroadaptation by alcohol is characterized by an abnormally AMG hyperactivity coupled with a hypofunction of the PFC and the HPC. This review describes research on molecular and epigenetic mechanisms by which alcohol causes distinct region-specific adaptive changes in gene expression patterns and ultimately leads to a variety of cognitive and behavioral impairments on prefrontal- and hippocampal-based tasks. Alcohol-induced neuroadaptations involve the dysregulation of numerous signaling cascades, leading to long-term changes in transcriptional profiles of genes, through the actions of transcription factors such as [cAMP response element-binding protein (CREB)] and chromatin remodeling due to posttranslational modifications of histone proteins. We describe the role of prefrontal–HPC–AMG circuit in mediating the effects of acute and chronic alcohol on learning and memory, and region-specific molecular and epigenetic mechanisms involved in this process. This review first discusses the importance of brain region-specific dysregulation of glucocorticoid concentration in the development of alcohol dependence and describes how persistently increased glucocorticoid levels in PFC may be involved in mediating working memory impairments and neuroadaptive changes during withdrawal from chronic alcohol intake. It then highlights the role of cAMP–PKA–CREB signaling cascade and histone acetylation within the PFC and limbic structures in alcohol-induced anxiety and behavioral impairments, and how an understanding of functional alterations of these pathways might lead to better treatments for neuropsychiatric disorders.

## Introduction

Alcoholism is a chronic, often relapsing brain disorder characterized by periods of sustained, compulsive alcohol intake, relying in part on allostatic changes within the prefrontal cortex (PFC) and limbic structures [i.e., the hippocampus (HPC) and the amygdala (AMG)] [for review, see Ref. (1)]. This circuit plays key roles in behavior and cognitive function as well as in orchestrating neuroadaptive responses to stress and anxiety. The transition from recreational to alcohol dependence and compulsive alcohol drinking takes place *via* neuroadaptive changes in the stress-related neural circuits, caused partly by repeated cycles of alcohol intoxication and withdrawal (2, 3). A prime mechanism that contributes to the development and maintenance of alcoholism is the dysregulation of the hypothalamic–pituitary–adrenal (HPA) axis activity (4) and the release of glucocorticoids (cortisol in humans and primates, corticosterone in rodents) from the adrenal glands. Clinical and preclinical evidence in both humans (5–7) and rodents (4, 8, 9) have shown that acute and chronic alcohol consumption, as well as withdrawal, markedly affects plasma glucocorticoid levels. The release of glucocorticoids can influence brain function by readily crossing the blood–brain barrier and exert effects through a dual glucocorticoid binding receptor system, i.e., the type I high affinity mineralocorticoid receptors (MRs) or the type II low affinity glucocorticoid receptors (GRs) (10), which act as ligand-dependent transcription factors to modulate target gene transcription. The MRs display a restricted expression in the brain, with highest densities in the HPC (11–13). The GRs are widely distributed throughout the brain (10, 14, 15) with a predominant expression in the three areas involved in learning and memory and particularly sensitive to the effects of stress, namely, the PFC, the dorsal HPC, and the AMG (16–18). Indeed, human studies of Cushing’s syndrome have shown that sustained cortisol elevation over the years compromises the integrity of the HPC–PFC circuitry and thus influences the onset and/or the severity of cognitive decline in various tasks, including spatial, decision-making and working memory processes (19–23). Further, sustained, high local concentration of glucocorticoids is responsible for long-lasting cognitive impairments occurring several weeks after the cessation of alcohol in rodents (24, 25) and abstinent patients (26, 27). As to how elevation of glucocorticoids might be implicated in the enduring cellular, molecular, and behavioral changes, it has been suggested that neuroadaptation induced by alcohol exposure involves the dysregulation of numerous signaling cascades, leading to long-term changes in transcriptional profiles of genes, through the actions of transcription factors such as [cAMP response element-binding protein (CREB)] and chromatin remodeling due to modifications of the posttranslational properties of histone proteins [for review, see Ref. (28)]. In the following, we provide an overview of how transcriptional and histone acetylation changes in the PFC, the HPC, and the AMG play a central role in the glucocorticoid-dependent neuroadaptation and behavioral deficits that occur during acute and chronic alcohol exposure. While this review focuses on aspects of how spatial and temporal changes in histone acetylation drive alcohol-induced alterations in neural plasticity and behavior, it should be emphasized that other histone modifications marks, such as histone phosphorylation and histone lysine methylation, occur in parallel and are also involved in the long-term adaptations in neural function and behavioral responses to alcohol exposure.

## Brain Regional Glucocorticoid Response to Chronic Alcohol Exposure

Surprisingly, little is known about the long-lasting neuroadaptive changes of glucocorticoids caused by prolonged alcohol consumption and withdrawal within neural circuits involved in learning and memory and emotional events and about their behavioral consequences. Studies, including our own, have shown that the initial phase of alcohol withdrawal period produces elevation in both circulating and brain glucocorticoids levels (29–31). Importantly, Little and colleagues (30) were first to show that during the initial phase of withdrawal from chronic (*8 months in rats*) alcohol consumption, rats and mice display an abnormal, exaggerated corticosterone level selectively in the medial PFC and the dorsal HPC. Strikingly, the authors found that withdrawal-associated excessive corticosterone response in the PFC persists for up to 2 months; therefore, long after, plasma corticosterone levels returned to baseline levels. In the PFC, the sustained elevation of corticosterone concentration was associated with enhanced GRs activation in mice undergoing a 2-week withdrawal period from chronic alcohol consumption (30). Further, administration of the GRs antagonist mifepristone or the dihydropyridine calcium channel nimodipine, given just prior to withdrawal from chronic alcohol exposure, not only reduced the rises in brain corticosterone but also prevented persistent memory deficits seen several weeks later in mice (24) or rats (32), suggesting that withdrawn-associated rise in glucocorticoid levels specifically within medial PFC may be an early index of maladaptive persistent behaviors in alcohol-dependent subjects. Indeed, chronic treatment with the GR antagonist mifepristone attenuated escalation of ethanol intake following intermittent ethanol vapor exposure (33) as well as the development of alcohol dependence and ultimately withdrawal-associated behavioral deficits (34). Endogenous glucocorticoids have been suggested to play an essential role in maintaining PFC-dependent cognitive functions, mainly *via* complex interaction with dopaminergic and glutamatergic receptors (35–37). Both human and animal studies have demonstrated that alcohol withdrawal impairs a variety of the cognitive functions during tests that require cortical prefrontal processing (38–40). As regards, pharmacological (hydrocortisone administration) or pathological (Cushing’s disease) increase of cortisol was found to predict frontal cortex-based cognitive impairments including alterations in executive processes and working memory dysfunction (19, 23, 41–43). Long-lasting deficits on tasks that rely on the PFC are also observed in rodent models in which chronic alcohol dependence is induced by chronic alcohol exposure or chronic intermittent ethanol that involves repeated cycles of exposure to alcohol vapors (44, 45). However, in addition to PFC dysfunction, there is evidence that a functional disconnection of brain network connectivity between the (dorsomedial) PFC and the central nucleus of the AMG may also contribute to the alcohol-induced working memory impairments in rats (46).

Recent work in our laboratory has employed *in vivo* microdialysis in freely moving mice to investigate effects of chronic alcohol treatment and withdrawal (early and prolonged) periods on brain corticosterone concentrations by simultaneously measuring time-course evolution of corticosterone concentration in the medial PFC and dorsal HPC seen before, during, and after completion of a working memory task in a T-maze (31, 47). This task is based on spontaneous alternation behavior, known to require intact connections between the two structures for successful performance (48, 49). Specifically, alternation behavior is the innate tendency of rodents to alternate at each successive trial the choice of the goal arm over a series of trials run in a T-maze (except for the first trial). From trial to trial, accurate performance at a given trial (*N*) requires for subjects to be able to discriminate the specific target trial *N* − 1 from the interfering trial *N* − 2. Thus, the target information required for successful performance varies from trial to trial, so that the subject is not only required to temporarily keep specific information in short-term storage but also reset it over successive runs. The resetting mechanisms and cognitive flexibility required to alternate over successive runs are major components of working memory processes. Working memory is a component of the sequential alternation task, since spontaneous alternation rates are dependent on the length of the inter-trial delay interval and/or the place of the trial in the series. Indeed, repetitive testing constitutes a potent source of proactive interference. Thus, the sequential alternation procedure is relevant to assess delay-dependent working memory in mice (50–52). Using *in vivo* microdialysis in freely moving mice, we observed that early (1 week) and protracted (6 weeks) withdrawal periods from prolonged (6 months) alcohol exposure causes an exaggerated corticosterone rise in the medial PFC. In addition, withdrawn mice having abnormal corticosterone concentration in the PFC displayed impaired working memory performance, effects that were not observed in animals still submitted to chronic alcohol consumption. Moreover, early and protracted withdrawal periods had no effect on the dynamic pattern of corticosterone response in the dorsal HPC, indicating that alcohol impacts glucocorticoid regulation in a brain region-specific fashion. During the 6-week withdrawal period, the degree of working memory impairment correlated with the magnitude of prefrontal corticosterone concentration, which is in accordance with the notion that there is a functional link between excessive corticosteroid signaling and PFC dysfunction (53–55). Many neuroimaging studies have indicated consistently that structural and functional deficits in PFC regulatory regions are associated with chronic alcoholism [for review, see Ref. (56)]. Another study using SPECT imaging showed that detoxified alcoholic patients who relapsed 2 months later displayed working memory deficits associated with low blood flow in the medial frontal lobe (57). Given the importance of frontal cortical regions in the modulation of AMG reactivity and the mediation of effective emotion regulation, weakened PFC function associated with a specific functional disconnection between the PFC and the AMG has been proposed as an early index of neuroadaptation in alcohol dependence that predicts PFC-dependent cognitive impairments observed during abstinence (38, 39, 46).

Subsequently, we have studied whether local glucocorticoid blockade in the medial PFC would prevent the long-term deficits in working memory induced by protracted withdrawal from chronic alcohol consumption (31). Intraperitoneal administration of the corticosterone synthesis inhibitor metyrapone prior to testing prevented the withdrawal-associated working memory impairments, confirming the essential role of persistently increased glucocorticoid levels in behavioral impairments during withdrawal from chronic alcohol intake. Similarly, a single bilateral infusion of spironolactone into the medial PFC that diminished MR activation and to a lesser extent of mifepristone that diminished GRs activation fully restored working memory function in withdrawn mice. In contrast, neither spironolactone nor mifepristone had any effect when infused into the dorsal HPC, thus highlighting the importance of glucocorticoids specific to the PFC in neural substrates mediating the prolonged, detrimental effects of alcohol on behavioral performance. These findings are reminiscent of data showing that elevated glucocorticoid levels, *via* either systemic injection of corticosterone or local infusion of the GRs agonist RU 28362 into the medial PFC shortly before testing, similarly impair working memory (55), while the GRs antagonist RU 38486 infused into the PFC can restore stress-induced deficits in executive function (58). Collectively, these data support the view that long-term adaptive behavioral effects of chronic alcohol exposure are mediated in large part through long-lasting glucocorticoid dysregulation within the PFC circuitry.

## Molecular Mechanisms Underlying Anxiety-Like and Alcohol-Drinking Behaviors: The Role of cAMP–PKA–CREB Cascade

The transcription factor CREB is a key downstream target of a variety of kinases, including cAMP–protein kinase A (PKA), Ca^2+^/calmodulin-dependent kinase, and extracellular-regulated kinase/mitogen-associated protein kinase (ERK/MAPK) (59, 60). The resulting activation/phosphorylation of CREB and recruitment of CREB-binding protein (CBP) along with other transcriptional components enables transcription of specific CREB target genes, including those implicated in long-term memory and plasticity as well as in the development of anxiety-like and alcohol-drinking behaviors, such as the neuropeptide Y (NPY) and the brain-derived neurotrophic factor (BDNF) (61–64). There is mounting evidence to support a role for phosphorylated CREB (pCREB) through a PKA-dependent mechanism and downstream CREB target genes, in the adaptive changes and behavioral effects associated with acute and chronic alcohol exposure [for review, see Ref. (65–68)]. Acute and chronic ethanol exposures have long been known to modulate the various steps of the cAMP-dependent pathways in the rodent brain and in other cell systems (69–71). Exposure to ethanol affects a cascade of events allowing for sustained translocation of PKA catalytic subunit into the nucleus (72), ultimately resulting in long-lasting increased CREB activation/phosphorylation (73) and downstream expression of many target genes (74). In this context, abnormal PKA-dependent CREB functioning has been implicated in the molecular mechanisms of neuroplasticity that underlie alcoholism and alcohol drinking. There is evidence for a biphasic temporal effect of ethanol on cAMP–PKA-dependent signaling cascade with acute and prolonged exposure to ethanol potentiating (75) and decreasing (76, 77), respectively, adenylyl cyclase–cAMP–PKA activity in the cortex and HPC (78) in mice. Using a combination of genetic or pharmacological approaches, *Drosophila* and rodents studies have shown that maintaining integrity of the cAMP–PKA activity is central to establishing sensitivity to the sedative effect of ethanol as well as in modulating ethanol consumption (79–81). Acute withdrawal (24 h) from chronic ethanol treatment produced a decrease in Ser133–pCREB within specific neurocircuitry of the frontal, parietal, and piriform cortex in rats (82), suggesting the possibility that CREB-dependent events in these cortical structures may be involved in the development of alcohol dependence. Among the mechanisms responsible for reduced pCREB and downregulation of cAMP-dependent genes, chronic intermittent alcohol exposure has been shown to increase expression of the protein kinase inhibitor-α (PKI-α) in the PFC, nucleus accumbens, and AMG in Wistar rats (83). Given the wealth of data for the recruitment of the cAMP–PKA signaling pathways upon acute ethanol exposure, it has been proposed that the increased PKI-α expression may be part of the adaptation of the cAMP–PKA pathway induced by intermittent alcohol exposure.

Investigations into the role of CREB in amygdaloid brain structures with regard to anxiety-like and alcohol-drinking behaviors have shown that CREB activity fluctuates depending on brain structures and alcohol “condition” (acute, chronic, or withdrawal). For instance, a series of studies by Pandey’s group conducted in the rat AMG clearly indicate a strong relationship between decreased CREB phosphorylation and high anxiety-like responses associated with acute withdrawal from 2-week ethanol treatment (62, 82). Decreases in CREB phosphorylation and downstream cAMP-inducible genes, including NPY in the central and medial, but not the basolateral, nuclei of the AMG, have been associated with a predisposition to both anxiety-like and excessive alcohol-drinking behaviors in alcohol-preferring rats (60, 84–86). Restoring CREB function to optimal level or enhancing NPY signaling in the central AMG prevented the onset of anxiety-like behaviors (84, 87, 88), while alcohol-associated anxiety disorders can be mimicked by pharmacological blockade of PKA in ethanol-naïve-preferring rats or non-preferring rats (60, 84). Thus, anxiety-induced downregulation of CREB function in the AMG may constitute a critical neuroadaptation central to the development and maintenance of alcohol dependence. As regards, dysregulation of the PFC associated with a functional disconnection between the PFC and AMG central nucleus during abstinence and renewed access to alcohol has been implicated in long-lasting cognitive impairment and excessive alcohol drinking in rats (46).

Clinical evidence from alcohol-dependent patients also indicates that acute and protracted withdrawal/abstinence is strongly associated with depressive-like behaviors, such as anhedonia.

The catecholamines dopamine and noradrenaline *via* the cAMP–PKA–CREB signaling cascade provide an essential modulatory influence on PFC-dependent behaviors producing an inverted “U-shaped” dose–response influence, whereby moderate levels improve PFC function while either too little or too much catecholamines lead to cognitive impairments [for review, see Ref. (89)]. A number of studies including work in our laboratory (51) have shown that blocking the cAMP–PKA–CREB signaling cascade *via* local infusion of Rp-cAMPS (a compound known to inhibit CREB phosphorylation) into the PFC prevents the impairing effect of stress or aging on working memory performance, while drugs that increase cAMP–PKA signaling either by direct intra-PFC infusion of the cAMP analog Sp-cAMPS or dopamine D1 receptor agonist or i.p. administration of the phosphodiesterase (PDE) inhibitor Rolipram impair cognitive functions [for reviews, see Ref. (89–91)]. As mentioned above, we recently reported that consumption of an alcohol-containing liquid diet for 6 months followed by a 1-week withdrawal period produces working memory impairment in a T-maze spontaneous alternation task in mice, which persists for at least 6 weeks after the cessation of alcohol intake (31, 47). Moreover, withdrawn mice displaying impaired working memory performance were those that had the lowest pCREB level in the PFC along with a persistent rise of prefrontal corticosterone concentration. Because glucocorticoids in the PFC interact with β-adrenoceptor–cAMP/PKA activity to influence working memory function (92), one route by which elevated glucocorticoid levels may impair PFC-mediated cognitive function long after the cessation of alcohol exposure is by inhibiting the cAMP–PKA cascade. In this context, growing evidence supports a central role for PDE, which is responsible for the breakdown of cAMP, in the regulation of alcohol drinking in rodents [for review, see Ref. (93)]. For example, treatment with various PDE4 inhibitors, including rolipram, produces long-lasting reduction of alcohol intake and preference in C57BL/6J mice (94). Chronic rolipram treatment also results in sustained reduction of alcohol seeking and consumption in alcohol-preferring rats (95, 96). As mentioned earlier, mice subjected to 1- or 6-week alcohol withdrawal from chronic alcohol consumption exhibited working memory impairments accompanied by enhanced anxiety level (at 1 week only) as well as persistently elevated corticosterone and sustained decreased pCREB levels in the PFC. Intraperitoneal administration of the PDE4 inhibitor, rolipram, before working memory testing abolished these withdrawal-associated behavioral, endocrine, and neuronal alterations (31) – a finding consistent with other observation, which demonstrated that in rats, heightened anxiety during acute alcohol withdrawal was accompanied by elevated expression of *Pde10a* isoform mRNA levels in interconnected medial PFC–AMG circuit, which persisted in the AMG after protracted (6 weeks) alcohol withdrawal (97). Together, these observations strongly support further research with regard to isoform-specific PDE-selective inhibitors that are promising pharmacotherapy targets for alcohol use disorders.

As discussed above, long-term adaptive behavioral effects of chronic alcohol exposure are mediated in large part through long-lasting glucocorticoid dysregulation within the PFC but not the dorsal HPC. Confirming differential sensitivity of the PFC and dorsal HPC to chronic alcohol-induced damage, recent work in our laboratory has shown that, unlike the PFC in which withdrawal from prolonged alcohol intake caused persistent working memory impairments along with sustained inhibition of the cAMP–PKA–CREB signaling cascade, both alcohol (unimpaired) and alcohol withdrawal (impaired) mice display reduced levels of pCREB in the dorsal HPC (namely, the CA1 region), compared with water-drinking mice (31, 47). Furthermore, intraperitoneal administration of rolipram was able to correct the deficit in pCREB in the dorsal HPC but did not reverse working memory impairments in withdrawn animals (47). Together, these observations support the notion that disruption of the cAMP–PKA–CREB signaling cascade specifically in the PFC (but not in the dorsal HPC) has an essential role in promoting long-term neuroadaptive changes accompanying persistent behavioral changes during withdrawal from chronic alcohol intake. Interestingly, early pioneering work in our laboratory emphasized a key role for PKA–CREB signaling as a sustained “molecular switch” that gradually converts acute “drug” responses into relatively stable adaptations that contribute to drug and alcohol addiction-mediated long-lasting neural and behavioral plasticity. Under conditions of drug- and food-reinforced behavior, drug-induced reward impaired spatial discrimination learning in a Y-maze task and caused drastic decreases in pCREB and downstream target c-Fos expression in the dorsal HPC and the PFC while sparing the cued version of the task and pCREB in the dorsal striatum in mice (98). Further, pharmacological blockade of cAMP–PKA cascade into the striatum before training normalized CREB activity within the HPC–PFC circuit and, as subsequently, prevented the drug-induced modulation of multiple memory systems.

Emerging evidence indicates that brain region-specific alteration of CREB signaling is also an important regulator involved in depression-like behavior that emerges during abstinence following alcohol drinking. As a key symptom of clinical depression, anhedonia reflects reduced interest in enjoying pleasure-seeking behavior and plays a key role in relapse (99, 100) and in the perpetuation of excessive alcohol consumption in dependent individuals (101). Important clinical evidence clearly demonstrated that the persistence and intensity of some behavioral withdrawal symptoms positively correlated with anhedonia scales in detoxified alcohol-dependent subjects (102), extending previous findings of strong correlation between anhedonia and substance-related symptoms particularly in detoxified opiate-dependent subjects (103). The presence of depression-related behavioral phenotypes during protracted abstinence was also reported in rodent models (104–106). In mice undergoing 2 weeks of abstinence from chronic alcohol consumption, the persistent increase in plasma corticosterone response and upregulation of GR expression correlated with the development of depressive-like phenotypes, including anhedonia and helplessness (105), and reduced hippocampal neurogenesis (104). Further, there are several lines of evidence that suggest that downregulation of BDNF–TrKB–CREB signaling pathway may serve as a common link between the development of alcohol-induced depression-like symptoms and reduced hippocampal neurogenesis (104, 105, 107, 108). Finally, since enhancing the BDNF–CREB activity through pharmacological treatments with various classes of antidepressant drugs or environmental enrichment abolished the alcohol-induced anhedonia and depressive behaviors seen during protracted abstinence (104, 107, 108), supporting the hypothesis that BDNF–CREB signaling pathway may be a potential therapeutic target for interventions in alcoholism–depression coincidence.

## Alcohol Alters the Balance Between Histone Acetylation: Deacetylation

Equally important for providing precise, long-lasting changes in brain function associated with alcohol intake are histone modifications, which exert lasting control over transcriptional activity of target genes through modifications of the chromatin structure and function that make the DNA less or more accessible to transcription factors and enzymes. The basic unit of chromatin, the nucleosome, is a histone octamer wrapped by approximately 147 base pairs of DNA. Each core histone (H2A, H2B, H3, and H4) has a highly conserved amino (N)-terminal tail, which is subject through a range of posttranslational modification (PTM) marks at distinct residues/sites including acetylation and methylation of lysine residues and phosphorylation of serine residues (109). Histones acetylation and phosphorylation are associated with transcriptional activation, whereas histone methylation reflects both transcriptional activation and repression depending on the specific site and context of the modification. An important feature of histone PTMs is that they can influence each other in a synergistic or antagonistic manner, leading to a complex “histone code” (110). Of these histone PTMs, histone acetylation is the most widely investigated in terms of epigenetic mechanisms underlying region-specific changes in brain gene networks required for long-term memory processes. Many rodent studies have detailed how different learning paradigms trigger distinct histone acetylation patterns in the brain, which are accompanied by region-, task-, and age-specific changes in memory-associated genes [for reviews, see Ref. (111–115)]. For instance, increased acetylation of histones, H3 and H4, occurred in the dorsal HPC or the dorsal striatum, depending on whether mice were subjected to a spatial or cued training in the water maze task, respectively (116, 117).

The degree of histone acetylation/deacetylation is finely orchestrated by dynamic balance of antagonistic enzymes that “write” (HATs) and “erase” [histone deacetylases (HDACs)] acetylation sites (113, 118–121). Systemic administration of HDAC inhibitors (HDACi), such as sodium butyrate (NaB) or trichostatin A (TSA), can improve memory formation and also prevent or reverse cognitive impairments associated with normal and pathological aging. However, this enhancing effect of HDACi on HPC-dependent memory required accurate CREB activity (116, 117, 122). Furthermore, infusing HDACi directly to the HPC was not only effective in promoting HPC-dependent learning and memory processes but can also influence relative use of multiple memory processes by affecting transcriptional events within subcortical and PFC cortical structures (116, 123).

A growing set of studies in both humans and animals have indicated that alcohol exposure causes widespread, dynamic changes of histone acetylation patterns, and thereby dysregulation in gene expression profiles across multiple brain regions (28, 124–126). Most of the studies have focused on the two histones H3 and H4 acetylation and chromatin-related events within the PFC, the HPC, and the AMG. In mouse and rat brain, studies reported that alcohol’s effects on histone acetylation patterns depend on the alcohol treatment paradigm, the timing of alcohol exposure or withdrawal, and brain structures examined, and even within a structure, alcohol can affect differently subregions. For example, work from Pandey’s lab has shown that anxiolytic-like responses caused by acute ethanol i.p. injection were accompanied by increased HAT CBP activity and associated increased acetylation of histone H3 at lysine 9 and histone H4 at lysine 8 (H3K9 and H4K8, respectively) leading to rapid elevation of NPY (mRNA and protein level) specifically in the central and medial, but not the basolateral amygdaloid, nuclei (125). The same group observed that a 2-week ethanol exposure followed by acute ethanol withdrawal (24 h) switches alcohol’s effect to anxiogenic-like responses, effects that involve a shift from HDAC hypoactivity to HDAC hyperactivity and subsequently decreased histone acetylation and transcriptional repression of NPY function in the two AMG nuclei (125, 127, 128). Correcting histone acetylation deficits in the AMG *via* administration of the pan HDACi TSA can reverse the rapid tolerance to the anxiolytic effects of ethanol (128) and prevent the development of alcohol withdrawal-related anxiety in rat (125). Alcohol-induced neuroadaptation in the AMG also implicated deficits of BDNF activity and its target [activity-regulated cytoskeleton-associated protein (Arc)], two key signaling factors involved in synaptic transmission and plasticity. While acute ethanol exposure caused an upregulation of BDNF–Arc signaling pathway and subsequently increased dendritic spine densities in the central and medial AMG nuclei, withdrawal from prolonged ethanol exposure or binge ethanol consumption potently inhibited BDNF and Arc expression and reduced dendritic arborization in these nuclei and other regions, leading to increased anxiety-like and drinking behaviors (66, 129, 130). Importantly, these long-lasting adaptive changes associated with alcohol dependence were reversed upon treatment with the HDACi TSA (61, 128–130). In another study by Moonat and colleagues (61) examining the role of HDAC2 in the development of alcohol dependence, investigators found lower baseline BDNF protein levels in the AMG (and also the bed nucleus of stria terminalis) of alcohol-preferring rats, a well-established model used to study the genetic predisposition to alcoholism (131), relative to the low-drinking NP rats. In addition, innate HDAC2 overexpression and decreased H3K9 acetylation in the central nucleus of alcohol-preferring rats correlated with low levels of BDNF, Arc, and NPY and was accompanied with high levels of anxiety-like and alcohol-drinking behaviors. These HDAC2-associated molecular and behavioral deficits were rescued *via* specific knockdown of HDAC2 expression either by direct infusion of small interfering RNA (siRNA) against HDAC2 into the central AMG nucleus (61, 66) or by TSA treatment (127, 130, 132). Collectively, these observations raised the possibility that adaptive epigenetic changes involving HDACs, and in particular HDAC2, in the AMG may be important regulatory mechanisms that underlie expression of genes implicated in the development and pathogenesis of alcohol dependence.

Using a chronic intermittent ethanol exposure model, a robust H3K9 hyperacetylation was seen in the AMG and cortical areas of rats, which displayed motivation to self-administer ethanol after a 6-h withdrawal period, compared with non-dependent rats (133). Treatment with the HDACi NaB or MS-275 (i.p. or i.c.v.) was able to counteract the effects of alcohol in dependent rats but not in non-dependent rats. Treatment with NaB, when administrated prior to ethanol self-administration, was also able to reverse H3K9 hyperacetylation and counteract excessive alcohol intake and relapse in alcohol-dependent rats. In order to identify brain region-specific regulatory molecular (*epigenetic*) signatures potentially involved in adaptive processes that lead to alcohol tolerance and dependence, Smith and colleagues (134) recently examined brain regional expression network responses to acute (0–8 h) and late (72 h to 7 days) withdrawal from chronic intermittent ethanol exposure in mice. Remarkably, the authors showed that neuroinflammatory responsive genes can be seen across all brain regions at 0–8 h after the beginning of alcohol withdrawal, while sustained over-representation for subset groups of genes related to neurodevelopment and synaptic plasticity (such as *Bdnf*) and to histone acetylation (such as *HDAC4* and *HDAC6*) and histone/DNA methylation are found at 3- to 7-day-withdrawal periods specifically in the PFC and the HPC. These results illustrate how transient and persistent histone acetylation changes could serve as a key mechanism for tight regulation of the expression of large sets of genes within specific brain regions of animals predisposed to excessive ethanol drinking or exposed to protracted abstinence. A functional disconnection of the CeA–PFC circuit during abstinence (72 h) and renewed access to alcohol has been recently implicated in long-lasting PFC-dependent cognitive dysfunction and the development of anxiety-like behavior, and more specifically, the resulting PFC hypofunction was shown to facilitate the transition from moderate to excessive and uncontrolled alcohol intake in rats (46).

Persistent changes of the HAT CBP activity and H4 acetylation were observed in the frontal cortex of C57BL/6 mice given 5-month chronic alcohol consumption followed by a 15-day withdrawal period (135). In that study, withdrawal-associated H4 hypoacetylation correlated with neuroinflammatory damage and the persistently altered memory and anxiety-related behaviors. Nonetheless, these changes were absent in mice lacking the Toll-like receptor 4 (TLR4) that have undergone the same treatment, suggesting a critical role for TLR4-mediated epigenetic modifications in mediating long-lasting deleterious effects of chronic alcohol on PFC-dependent behaviors (135). This is in line with findings in our laboratory showing a robust decrease in histone H4 acetylation in the medial PFC of C57/BL mice at 1 week after withdrawal from chronic alcohol consumption; this decrease was maintained for at least 6 weeks after alcohol withdrawal and correlated with the persistently impairment of working memory noted during abstinence (31, 47). Alcohol’s effects on H4 acetylation closely paralleled effects on CREB activation in the PFC. Further, systemic delivery of corticosterone inhibitor metyrapone or local intra-PFC blockade of MRs (*via* spironolactone) or GRs (*via* mifepristone) similarly reversed long-lasting deficits in pCREB and H4 acetylation levels in the PFC and alleviated working memory deficits associated with alcohol withdrawal (31). Thus, these findings suggest that long-lasting glucocorticoid-induced neuroadaptive changes in CREB and H4 acetylation in the PFC may be involved in the enduring working memory impairments caused by prolonged alcohol consumption and withdrawal. Cumulative evidence indicates that structural and functional integrity of the HPC was also compromised in rats after prolonged alcohol exposure and even greatest alterations were found after cessation of alcohol exposure (136–138). Prolonged ethanol intake caused enduring deficits in HPC-dependent spatial reference memory in the water maze (138–140). Chronic ethanol treatment also caused long-lasting decrease of histone acetylation in the dorsal HPC. However, contrary to the PFC where there was strong relationship between alcohol-induced decrease of H4 acetylation and long-lasting working memory impairments, H4 acetylation in the HPC (*the CA1 region*) was decreased in behaviorally “unimpaired” alcohol-treated mice and even continued to decrease in “impaired” withdrawal-treated mice, compared with water-treated mice (31, 47). However, the drugs that prevented alcohol’s effects in the PFC did not rescue alcohol’s effects on HPC function, underscoring a region-specific influence of regulatory epigenetic signature on adaptive processes that lead to alcohol tolerance and dependence.

## Alcohol and Histone H3 Modification Cross Talks

Ethanol’s effects on histone H3 phosphorylation at serine 10 (H3ser10phos) and concurrent H3 phosphoacetylation are of particular interest as their rapid elevation is critical for leaning/memory-associated induction of immediate-early genes (e.g., *c-fos* and *egr-1*) (141–143), an effect shown to mediate adaptive responses to psychology stressful events such as forced swimming or novelty stress paradigm exposure (142–145). In rats, acute ethanol dose dependently alters the number of H3ser10phos in the dentate granular cells of the HPC, and these changes are paralleled by changes in c-fos protein expression (146). The same group has shown that, in ethanol-dependent rats, both H3ser10phos and c-fos levels are reduced in dentate granule cells during excessive alcohol intake, while opposite effects are evident at withdrawal peak in the HPC. Elevation of H3ser10phos and histone H3 phosphoacetylation is achieved through a direct interaction of the GR with [mitogen- and stress-activated protein kinase 1 (MSK1)] and ETS-domain protein Elk-1 that are downstream of the ERK/MAPK signaling cascade (143, 145, 147, 148). Conversely, nuclear type 1 protein phosphatase (PP1), a nuclear protein Ser/Thr phosphatase that acts as a universal negative regulator of memory and synaptic plasticity, interfered with H3Ser10phos in several brain areas such as the HPC and the AMG (149–152).

Combinatorial modifications of acetylated H3 and histone H3 lysine 4 trimethylation (H3K4me3) have been implicated in long-term adaptive changes in the HPC resulting from prolonged alcohol intake (126, 153). Using a 3-week mouse model of chronic ethanol consumption, Stragier and colleagues (154) recently reported that ethanol-induced BDNF-mediated neuroplastic changes in the HPC are controlled by combinatorial modifications of acetylated H3 and H3K4me3 around individual *Bdnf* gene promoters in dorsal CA3 region and the dentate gyrus and by decreased *Bdnf* DNA methylation in CA1–CA3 regions of the HPC. These ethanol-induced changes were associated with a deficit in HPC-dependent (contextual fear and novel recognition object) memory while sparing AMG-based cued fear memory. Chronic intermittent ethanol vapor exposure followed by 2–5 days of abstinence robustly and selectively increased histone H3K9 acetylation and DNA demethylation in PFC neurons with a parallel decrease of H3K9 methylation repressive mark as well as a downregulation of a set of histone methyltransferases (HMT) (155, 156). These changes mostly occurred after ethanol removal and contributed to the development of physical dependence on alcohol through an adaptive long-lasting upregulation of the NMDA receptor 2B (NR2B) gene expression (155). Moreover, systemic treatment with TSA during ethanol exposure increased H3K9 acetylation at the NR2B promoter in PFC neurons and potentiated voluntary ethanol consumption (157). Together, these data suggest that persistent upregulation of the NR2B-containing NMDA receptors through deregulation of the balance between histone H3K9 acetylation and methylation states in the PFC may act as a potentially important contributor to the development of alcohol dependence.

## Concluding Remarks

**Figure 1:**
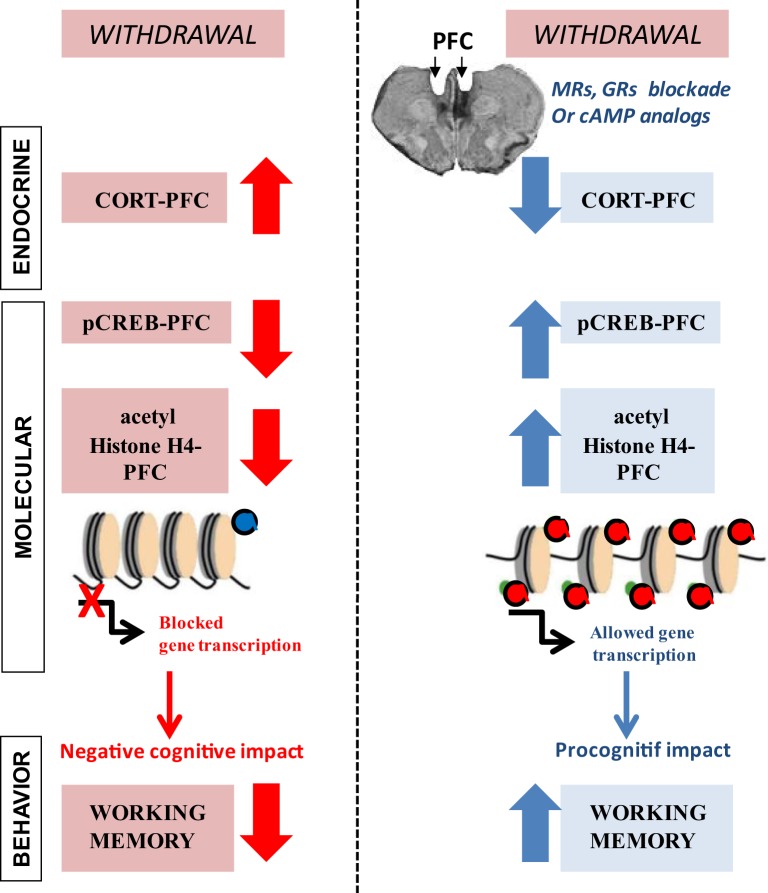
**Endocrine and molecular events that are associated with long-lasting prefrontal cortex (PFC)-based working memory impairment during withdrawal from prolonged ethanol consumption**. (Left) in C57BL/6 mice, chronic ethanol consumption (6 months) followed by a 6-week withdrawal period causes excessive peak corticosterone (CORT) response, specifically in the PFC that lasts for several weeks. Protracted withdrawal also produces long-lasting deficits in pCREB and histone H4 acetylation levels in the PFC along with enduring working memory impairments. (Red) pharmacological glucocorticoid blockade in the PFC through bilateral infusion of drugs that diminish corticosteroid receptors [mineralocorticoid (spironolactone; MR) or glucocorticoid (mifepristone; GR)] activity as well as pharmacological elevation of cAMP–PKA-mediated signaling cascade through bilateral infusion of the cAMP analog Sp-cAMPS into the PFC fully prevent long-lasting alcohol-related endocrine, molecular, and behavioral changes. Adapted from Ref. (31, 47).

This review summarizes recent advances in our comprehension of endocrine, epigenetic, and transcriptional changes that serve as determining factors in controlling alcohol-associated changes in the expression of gene networks and behavior and play a central role in the regulation of alcohol dependence, withdrawal, and relapse (Figure 1). Most of the studies conducted thus far focused mainly on epigenetic and transcriptional regulation of adaptive responses to acute and chronic alcohol that occur within a single brain region (mostly the AMG). This review highlights new evidence from clinical and preclinical studies on how long-term adaptations arising from disruption of the fine coordination of highly interconnected brain structures within a circuit, including, but not limited to, the PFC, the HPC, and the AMG, may contribute to excessive alcohol consumption and alcohol dependence as well as behavior impairments. The findings reviewed in this article support the view that brain region- and cell type-specific histone acetylation modification (both in terms of global/genome-wide changes as well as promoter-specific changes) is a key mechanism underlying anxiety-like and alcohol-drinking behaviors. Thus, treatments designed to counteract alcohol-associated epigenetic changes may be promising targets for novel medications in the treatment of alcoholism.

## Author Contributions

NM and DB contributed to the writing of this review article.

## Conflict of Interest Statement

The authors declare that the research was conducted in the absence of any commercial or financial relationships that could be construed as a potential conflict of interest.
